# Spatio-temporal pattern of short birth interval and associated factors on women in Ethiopia: Using Ethiopian demographic and health surveys 2000–2016

**DOI:** 10.3389/fmed.2023.1131794

**Published:** 2023-04-06

**Authors:** Gezachew G. Arega, Aweke A. Mitku, Haile M. Fenta

**Affiliations:** Department of Statistics, College of Science and Maritime Academy, Bahir Dar University, Bahir Dar, Ethiopia

**Keywords:** autologistics spatial model, autocorrelation, hotspot analysis, short birth interval, spatial

## Abstract

**Background:**

A short birth interval is a critical factor that contributes to a large number of maternal and infant mortality in low- and middle-income countries. It is the major cause of maternal and child mortality in Ethiopia. This study aimed to explore the spatiotemporal distribution of short birth intervals in Ethiopia using data from four (2000, 2005, 2011, and 2016) consecutive demographic and health surveys.

**Methods:**

A total of 34,930 women were included in four consecutive Ethiopian Demographic and Health Surveys (EDHS). Thus, spatial autocorrelation, hotspot analysis, cluster analysis, and spatial interpolation were carried out for each survey separately to show the geographical and temporal pattern of at-risk areas for short birth intervals in Ethiopia. Finally, the highest proportion of short birth interval risk areas in each survey period was mapped. Geospatial analysis was conducted by using ArcGIS V.10.8 and R version 4.2.

**Results:**

The results of the study indicated that the overall proportion of short birth intervals of women in Ethiopia was highest in 2000 (47.5%), 2005 (46.4%), 2011 (44.7%), and the lowest in 2016 (44.0%). The values for Global Moran’s I (MI = 0.177665 *p* = 0.0016, MI = 0.2024, *p* = 0.001, MI = 0.10023, *p* = 0.002, and MI = 0.764, *p* = 0.008) showed that the presence of significant short birth interval clustering in Ethiopian administrative zones in 2000, 2005, 2011, and 2016, respectively. The hotspot areas for short birth intervals were consistently observed in the zones in the Somali Region and the zones in the Harari Region for all the EDHS years. In addition, the survival status of the index child, residence, breastfeeding practice, religion, and the spatial variable (Si) were significantly associated with the short birth interval of women in all the EDHS years.

**Conclusion:**

Spatial distribution of short birth intervals differs across Ethiopian administrative zones. Survival status of the index child being dead, rural residential, and no breastfeeding practice are the risk factors for short birth intervals of women that increase the risk of a short birth interval among women in all the EDHS years. Therefore, the hotspot areas and indicators need interventions to decrease the short birth interval of women.

## 1. Introduction

High fertility rates promote population growth and undermine efforts to develop all areas of civilization, in addition to high levels of closely spaced and unwanted births. Births that are closely spaced have the potential to be harmful to both the individual and society. This problem, along with high rates of unwanted pregnancy, makes it challenging for women to participate in society and lowers their economic contribution (1).

The time between the birth of the child under study and the birth of the child immediately before it is referred to as a preceding birth interval. In order to reduce the risk of negative effects on the health of both the mother and the child, the World Health Organization’s (WHO) most recent recommendation for a healthy pregnancy gap is at least 2 years, or a birth-to-birth interval of 33 months, assuming 9 months gestation. The term “optimal spacing until the next pregnancy” refers to the interval between pregnancies during which the mother can recuperate from pregnancy, childbirth, and nursing. The next pregnancy and birth are more likely to occur at full pregnancy and growth when there is a long gap between them (2). A short birth interval (SBI) is defined as a period between two consecutive live births of less than 33 months (33 months = 24 months from birth to conception +9 months of pregnancy). Non-short birth intervals were defined as preceding birth intervals exceeding 33 months, which is in line with WHO recommendations (3).

Preterm birth, small for gestational age, and low birth weight are common in babies born during short birth intervals, while their mothers may experience maternal anemia, fetal loss, premature rupture of membranes, and eclampsia. As a result, not only is good birth spacing vital for population control, but it is also important for promoting mother and child health (4).

A short birth interval is a critical factor that contributes to a substantial number of maternal and perinatal mortality in low- and middle-income countries. Around the world, there are thought to be 2.6 million stillbirths each year. Optimizing birth intervals can prevent the majority of stillbirths that happen during the intrapartum period, with Sub-Saharan Africa, which includes Ethiopia, accounting for 67% of all stillbirths worldwide. Birth intervals that are too short (less than 18–27 months) or too lengthy (usually more than 54–59 months) are related to poor health outcomes for both mothers and children (5).

In underdeveloped nations, women experience shorter childbirth intervals. Short birth intervals are primarily caused by the fact that many women in developing countries do not take contraception after giving birth, increasing their chances of becoming pregnant once their fertility returns. By spacing births, contraception is a successful method of controlling fertility and improving mother and child health (6, 7).

Ethiopia is the second-most populous country in Africa with a population of more than 100 million and a fertility rate of 4.6 children per woman. Like many other African countries, Ethiopia has seen minimal improvement in the lowering of fertility due to socio-cultural and religious considerations (8). Short birth intervals have an impact on fertility, as well as neonatal, infant, and childhood mortality in Ethiopia, Infant mortality is reduced by 50% and fertility is decreased by 43% when the birth interval is extended by at least 2 years (9).

In this study, spatial covariates, global autocorrelation, and cluster analysis were utilized to identify spatial autocorrelation. After that, an autologistics spatial regression model was estimated and a diagnostic test was run to see if the variables were adequately represented to reflect the spatial dependency of the dependent variable. When data for geographical factors are available, spatial autologistics models describe SBI variation by geographical location better than non-spatial models. Highlighted that geographically situated data analysis is one of the statistician’s key concerns, and as a result, it is becoming increasingly significant in other disciplines of research. Tests of spatial autocorrelation tests are used to determine the level of clustering and to make statistical inferences (10).

## 2. Methods

### 2.1. Study design, period, and setting

A cross-sectional survey study design was conducted in Ethiopia using the 2000, 2005, 2011, and 2016 Ethiopian Demographic and Health Surveys (EDHS). Ethiopia is located in the Horn of Africa at (3°–14° N and 33°–48°E). The country has a surface area of 1.1 million square kilometers with a high central plateau that ranges in altitude from 4,550 m above sea level to 110 m below sea level in the Afar Depression. The country has 9 National Regional States namely Tigray, Afar, Amhara, Oromia, Somali, Benishangul Gumuz, Southern Nations, Nationalities and People’s Region (SNNPR), Gambella, and Harari, and two administrative cities, namely Addis Ababa and Dire Dawa, and the regions are divided into 74 zones.

### 2.2. Source and study population

The source population was all women of reproductive age (15–49) in Ethiopia. The study population was reproductive-age women who gave live birth in the 5 years preceding the survey.

#### 2.2.1. Sample size and sampling procedure

**Figure 1:**
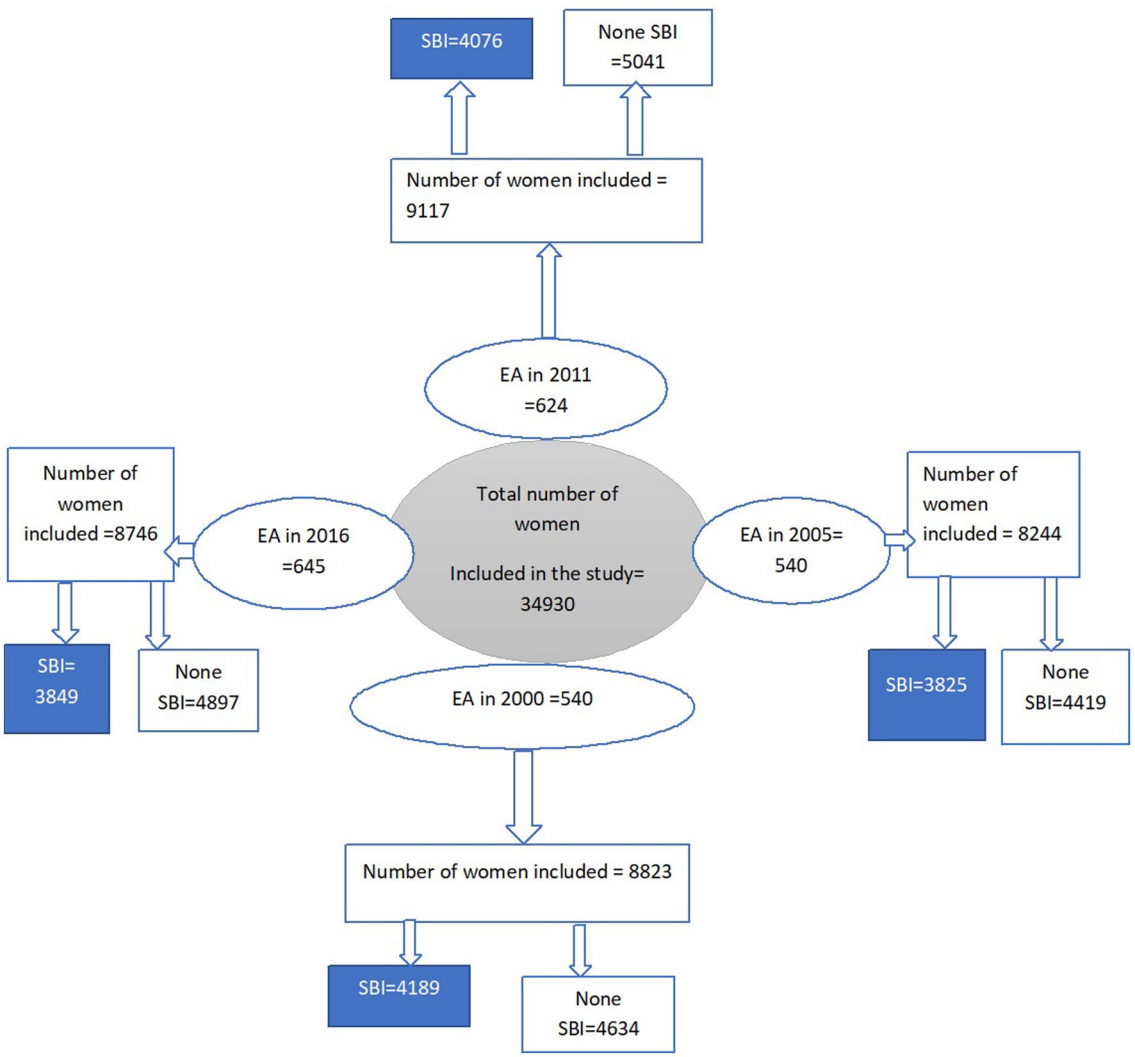
The flowchart for the data extraction procedure from EDHS 2000 to 2016, Ethiopia.

In this study, a weighted sample size of 34,930 (8,823 in 2000, 8,244 in 2005, 9,117 in 2011, and 8,746 in 2016) individuals with the most recent birth were included in the analysis (Figure 1). Weighted values were used to restore the representativeness of the sample data. Participants were selected based on a stratified two-stage cluster sampling technique in each survey year (2000, 2005, 2011, and 2016). The detailed sampling procedure was available in each EDHS report from the DHS website.^1^

The EDHS used a two-stage sampling method to select households and individuals to participate in the survey. In the first stage, a stratified cluster sampling method was used to select sample enumeration areas (EAs) from a list of all EAs in the country. EAs are geographic units consisting of a certain number of households, and they are defined by the Central Statistical Agency (CSA) of Ethiopia. The selection of EAs was stratified by region and urban–rural residence, and a systematic random sampling method was used to select EAs from each stratum.

In the second stage, a fixed number of households were selected from each EA. This was done using an equal probability systematic selection method, where a household was chosen at random from a list of all households in the EA. All eligible individuals in the selected household were then invited to participate in the survey.

#### 2.2.2. Data collection tools and procedures

When this work was requested and the www.dhsprogram.com website was accessed, the data was obtained from the Demographic and Health Surveys (DHS) Program. Data from the EDHS were gathered using stratified two-stage sampling. The country’s regions were divided into urban and rural areas. Outliers or erroneous data may occur in any data collection process, including the EDHS. However, the EDHS implemented various measures to minimize such occurrences and ensure the accuracy of the data. The survey used a standardized methodology and questionnaire, and data collectors received extensive training to minimize errors in data collection. Additionally, field supervision and quality control measures were in place to monitor the data collection process and identify any potential issues. EDHS also conducted data quality checks and data cleaning procedures to identify and correct errors and inconsistencies in the data. Overall, the EDHS has undergone rigorous quality control processes to ensure the accuracy and reliability of the data.

### 2.3. Variables

#### 2.3.1. Outcome variable

The outcome variable of this study was the SBI of women. This was a binary outcome variable coded as 1 = short birth interval of women and 0 = not.

#### 2.3.2. Independent variables

From the EDHS dataset, demographic and socio-economic, reproductive, behavioral, and child status variables were taken as the independent variables in the four consecutive survey years.

#### 2.3.3. Operational definition

“Index child” generally refers to the first child in a family who is diagnosed with a particular medical condition or disease. For example, if a woman has a child with a short birth interval, that child would be considered the index child.

### 2.4. Statistical models and analysis

The data is managed by STATA version 16 software and SPSS version 26. Sample weighting was done before further analysis.

#### 2.4.1. Spatial autocorrelation

For spatial autocorrelation and the identification of hot spot zones, we used Arc GIS 10.8 software. The spatial autocorrelation statistic (Global Moran’s I) was used to determine whether SBI was dispersed, clustered, or randomly distributed in Ethiopia. A Moran’s I value close to −1 indicates dispersed SBI, close to +1 indicates clustered, and a Moran’s I value of zero indicates randomly distributed SBI (11).

#### 2.4.2. Hot spot analysis

The proportion of SBI in each Ethiopian administrative zone was taken as input to analyze the hotspot analysis. Hot spot analysis (using the Hot Spot Analysis tool to calculate the Getis-Ord Gi* statistic) of the z-scores and significant *p*-values indicates either hot spot or cold spot values (12). A hotspot refers to the occurrence of a high proportion of SBI clustered together on the map, whereas a cold spot refers to the occurrence of a low proportion of SBI clustered together on the map.

#### 2.4.3. Spatial interpolation

The spatial interpolation technique was used to predict the SBI of women for unsampled areas based on sampled areas. For the prediction of unsampled areas, we used a geostatistical ordinary Kriging spatial interpolation technique using ArcGIS 10.8 software.

#### 2.4.4. Autologistic regression model

One of the most popular models for modeling spatially linked binary (yes/no) data is the autologistic regression model. Recent studies have shown that the autologistic regression model performs well when combined with autocovariate variables to model binary data with observable covariates. The autologistic regression model is a special case of the generalized logistic regression model. The model introduces spatial autocorrelation terms in the form of weighting coefficients and solves the problem of spatial autocorrelation effects in the process of statistical analysis (13). We can express the probability of the occurrence of SBI using the equation:


$$\pi_{i}=\frac{\exp\left(x_{i}^{\prime }\beta +r\;{\mathrm{Autocov}}_{i}\right)}{1+\exp\left(x_{i}^{\prime }\beta +r\mathrm{autocovi}\right)}$$

Where $\mathrm{Auto}\;{\mathrm{cov}}_{i}=\frac{\sum_{j=1}^{ki}w_{ij}p_{j}}{\sum_{j=1}^{ki}w_{ij}}$ .

The predicted result $\pi_{i}$ denotes the probability of an event occurring for every zone x and is the independent variable. Auto ${\mathrm{cov}}_{i}$ is the auto covariate variable. β and r are the coefficients of the explanatory variable and the coefficient of the fixed autocovariate variable. In the equation, i is the index of the geographical zone and $w_{ij}$ is queen weight matrix.

Spatial autocorrelation is frequently measured in spatial data. Spatial risk factors for diseases are often strongly auto-correlated, which means that two similar units that are close together in space likely to have more in common than would be predicted by chance. As a result, models that ignore spatial autocorrelation may not be appropriate since they could give an environmental variable too much weight. Additionally, by including variables that were important to the response variable in models that account for the spatial autocorrelation effect, it was possible to draw accurate inferences about the spatial distribution of SBI. By using spatial autocorrelation (autocovariate) in logistic regression models, it may be possible to resolve this problem while also improving the models’ variability and precision. By adding an autocovariate variable that is derived from the binary logistic regression model is changed to the autologistics regression model, which incorporates any spatial autocorrelation between geographic units (13, 14).

The autocovariate variable can be calculated from the predicted probabilities of occurrence, which is estimated by the binary logistic regression model using this equation.


$$\mathrm{Auto}\;{\mathrm{cov}}_{i}=\frac{\sum_{j=1}^{ki}w_{ij}p_{j}}{\sum_{j=1}^{ki}w_{ij}}$$

The autocovariate variable (𝐴𝑢𝑡𝑜𝑐𝑜𝑣𝑖) is the weighted average of the probability of the geographical units among a set 𝑘𝑖 neighbors of the geographical unit *i*, a method with a certain distance. Assume similarly (neighbored) of the centroid is used to define the neighbors of the geographic unit *i* in the study area, and $w_{ij}$ is queen weight matrix and $p_{ij}$ represents the predicted probability estimated by the binary logistics regression model.

## 3. Results

The study results showed that, among the weighed sample of 15,939 individuals, 84%, 81%, 75%, and 74% of the SBI were observed from uneducated mothers in 2000, 2005, 2011, and 2016, respectively. From the total of SBI, 88.3%, 90.4%, 87.7%, and 89.3% were observed in rural areas, and 11.7%, 9.6%, 12.3%, and 10.7% were observed in urban areas in 2000, 2005, 2011, and 2016, respectively. Among the total number of women who had an SBI, 91.8%, 86.9%, 73.9%, and 70.7% were women who did not use a contraceptive method, and 8.2%,13.1, 26.1%, and 29.3% were women who did use a contraceptive method in 2000, 2005, 2011, and 2016, respectively (Table 1).

**Table 1:** Factors that affect short birth intervals.

Variables	Category	SBI				Non-SBI			
		2000	2005	2011	2016	2000	2005	2011	2006
Mother Education	No education	3534(84.3%)	3081(80.6%)	3080(75.6%)	2860(74.3%)	3957(85.4%)	3578(81%)	3506(69.5%)	3360(68.6%)
	Primary	482(11.5%)	572(15%)	882(21.6%)	829(21.5%)	471(10.2%)	580(13.1%)	1282(25.4%)	1195(24.4%)
	Secondary	158(3.8%)	149(3.9%)	62(1.5)	110(2.9%)	219(59.5%)	219(5%)	145(2.9%)	211(4.3%)
	Higher	16(0.4%)	22(0.6%)	51(1.3%)	50(1.3%)	42(65.6%)	42(1%)	108(2.1%)	131(2.7%)
Residence	Urban	492(11.7%)	368(9.6%)	503(12.3%)	412(10.7%)	640(13.8)	539(12.2%)	997(19.8%)	842(17.2%)
	Rural	3697(88.3%)	3456(90.4%)	3573(4687.7%)	3437(89.1%)	3994(86.2%)	3881(87.8%)	4043(80.2%)	4056(82.8%)
Contraceptive use	No	3844(91.8%)	3324(86.9%)	3012(73.9%)	2720(70.7%)	4287(92.5%)	3800(86%)	3719(73.8%)	3142(64.1%)
	Yes	345(8.2%)	501(13.1%)	1063(26.1%)	1129(29.3%)	347(7.5%)	619(14%)	1322(26.2%)	1756(35.9%)

SBI, short birth interval; Non-SBI, non-short birth.

The spatial distribution of SBI in Ethiopian administrative zones was non-random among the four consecutive surveys. The global Moran’s I value was 0.177665 (*p*-value 0.0016) in 2000, 0.2024 (*p*-value < 0.0015) in 2005, 0.10023 (*p*-value 0.002) in 2011, and 0.764 (*p*-value < 0.001) in 2016 (Table 2).

**Table 2:** Summary results of global Moran’s I.

EDHS survey year	2000	2005	2011	2016
Observed Moran’s I	0.177665	0.2024	0.10023	0.764
Z-score	3.1368	3.79	2.9021	3.92598
*P*-value	0.0016	0.0015	0.002	0.0008

**Figure 2:**
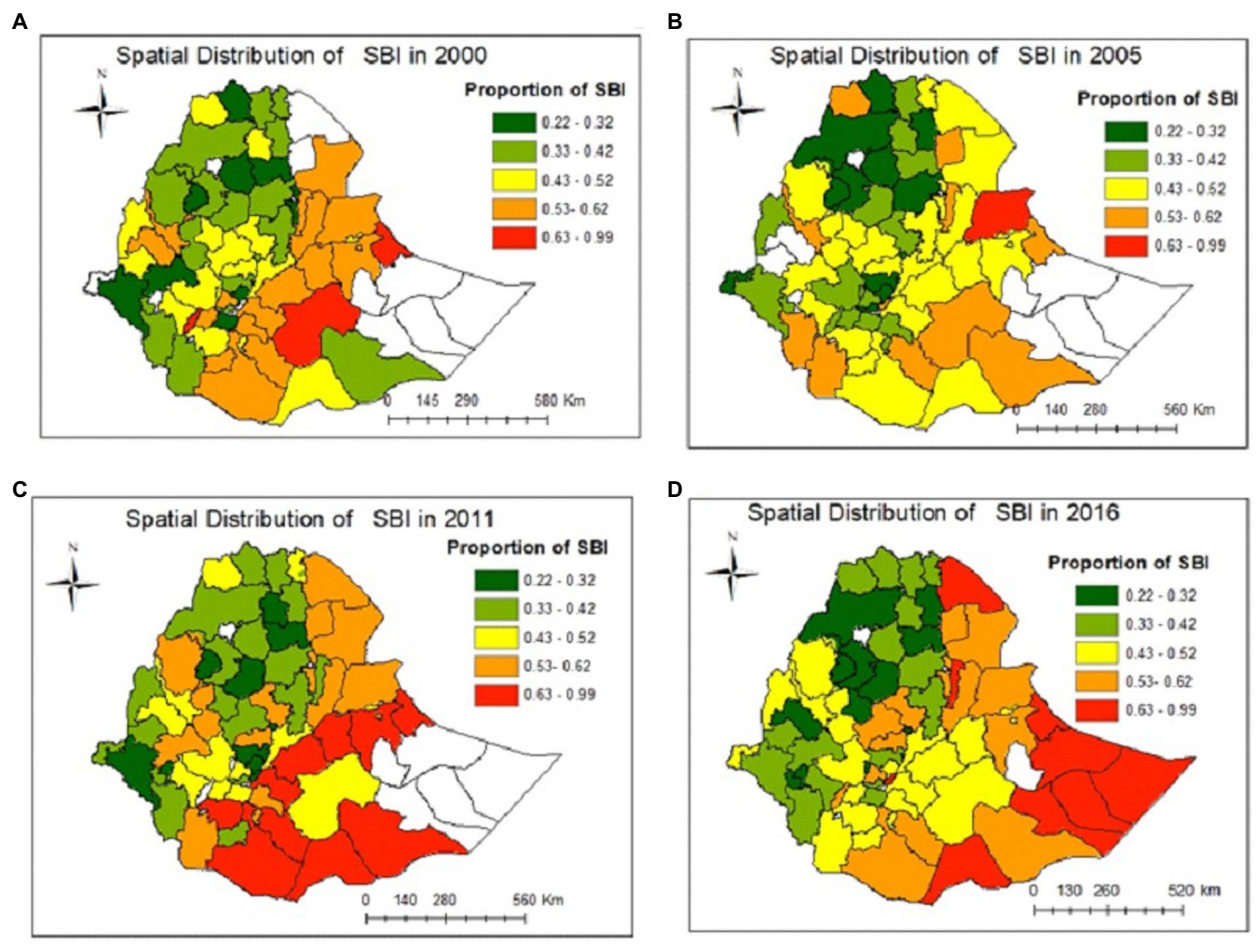
Observed spatial distribution and temporal pattern of short birth intervals in Ethiopia in the years **(A)** 2000, **(B)** 2005, **(C)** 2011, and **(D)** 2016.

The spatial distribution of SBI in Ethiopian administrative zones was presented in Figure 2. The maps showed that the proportion of short birth intervals in each administrative zone varied significantly over time. The red color indicates a high proportion of SBI and the green color indicates a low proportion of SBI. A high proportion of SBI was consistently observed in the zones of Fafan and Sitti in Somali, the western zone of the Harari Region, and the Guji zone in the Oromia Region in all the EDH years. A high proportion of SBI was consistently observed in Zone 5 in the Afar Region in 2000, 2005, and 2016 and Zone 4 in the Afar Region in 2005, 2011, and 2016. Similarly, a high proportion of SBI was consistently observed in the zone of Borona (Oromia region) in 2000, 2011, and 2016 and in western and eastern Harerge in 2000 and 2016. In addition, some zones fluctuated which indicated that the proportion of SBI changed over time and the white (colorless) zone indicated that there is no enumeration area or data about SBI at that zone (Figure 2).

### 3.1. Hotspot analysis of SBI in four survey years

**Figure 3:**
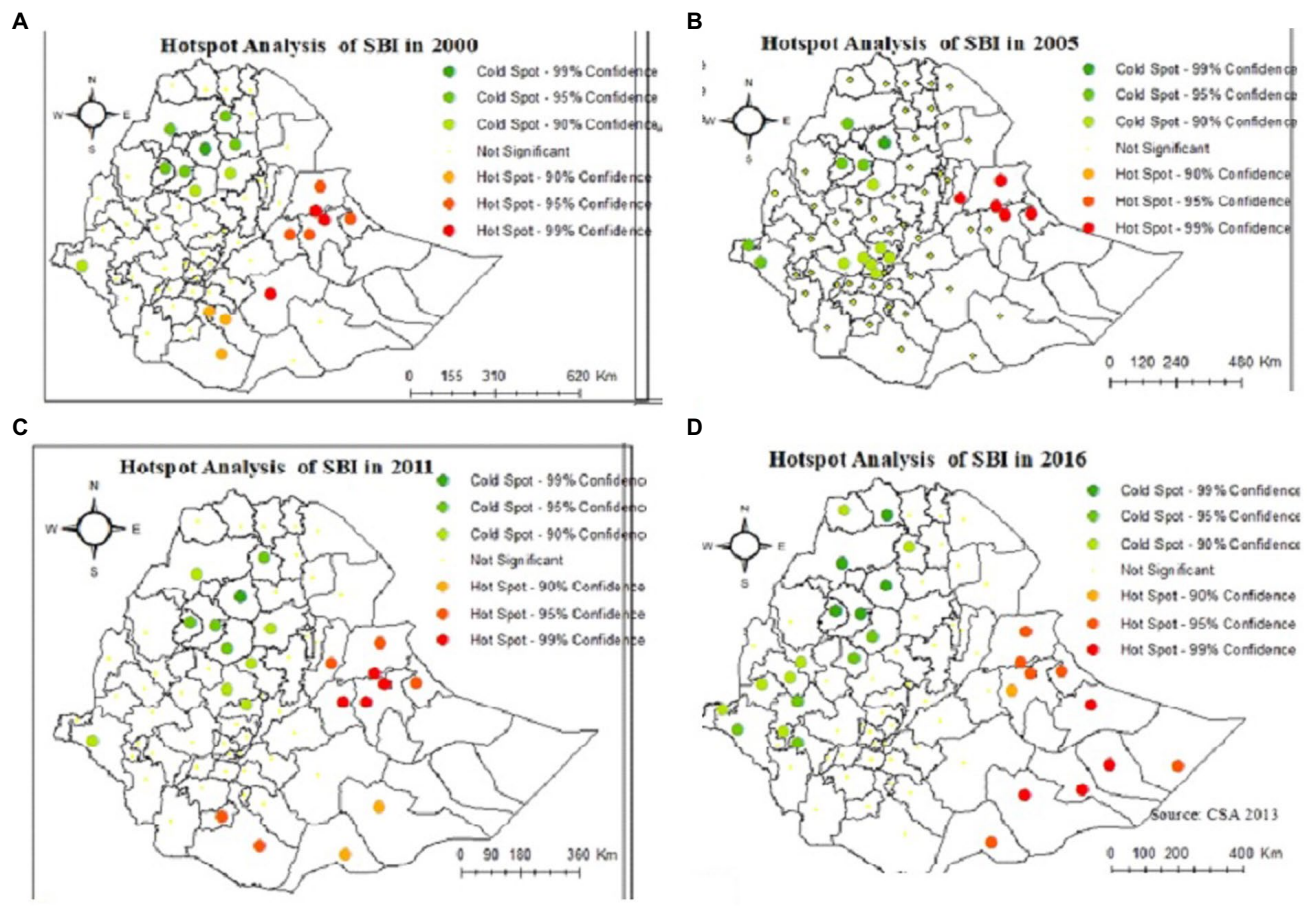
Hot spot and cold spot administrative zones of short birth intervals in Ethiopian in the years **(A)** 2000, **(B)** 2005, **(C)** 2011, and **(D)** 2016.

In the hot spot (Getis-Ord Gi*) statistics, significant hot spot and cold spot areas of SBI were identified. As shown in Figure 3, the red color indicates significant hot spot (high-risk) areas for SBI at 95 and 99% confidence intervals. The green color indicates the cold spot (low-risk) areas for SBI at 95% and 99% confidence intervals. A hotspot of SBI at 95% and 99% confidence interval was consistently observed in the Fafan zone in the Somali Region and the Harari and East Harerge zones of Harari Region for all the survey years.

Kriging interpolation was employed to explore the burdens of SBI on women in the unsampled areas of the country based on the observed data. The spatial interpolation technique was used to predict SBI in the un-sampled areas in the country based on the value observed from sampled areas. Therefore, part of a certain area can be predicted by using observed data using a method called interpolation. There are various deterministic and geostatistical interpolation methods. Among all of the methods, ordinary kriging and empirical Bayesian kriging are considered the best methods since they incorporate the weighting (15). In this study, the ordinary kriging spatial interpolation method was used for the prediction of SBI in unobserved areas of Ethiopia since it had the lowest residual.

### 3.2. Spatial distribution of autocovariate variable

**Figure 4:**
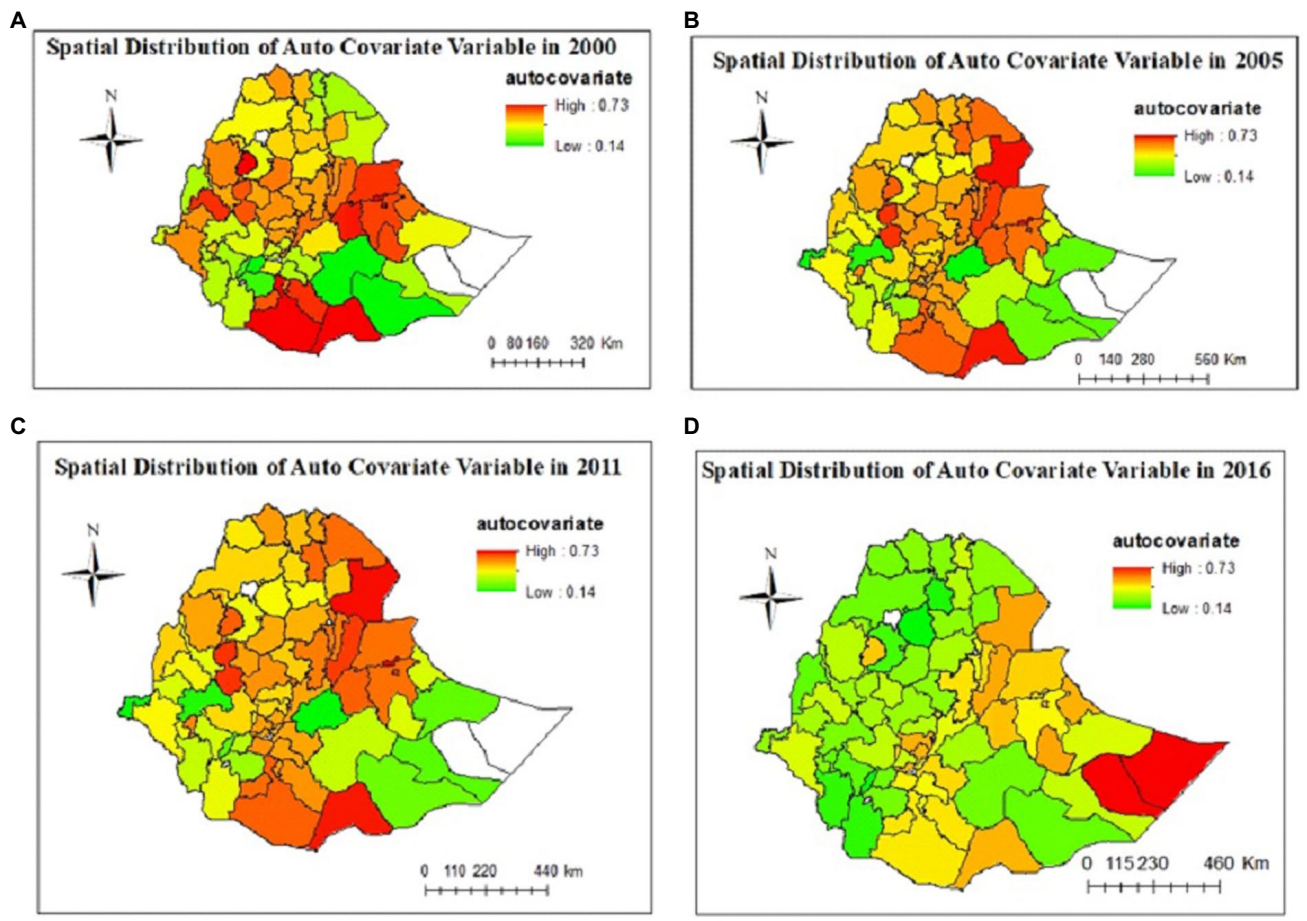
Predicted spatial effects of SBI in Ethiopian administrative zones and temporal trend in years **(A)** 2000, **(B)** 2005, **(C)** 2011, and **(D)** 2016.

Spatial autocorrelation (auto covariate) of malaria is almost predictable as human populations -live in spatial clusters rather than in random distributions of regions. Announcing the spatial autocovariate variable reflects spatial autocorrelation. It is a process of data smoothing, reducing local spatial dependence between geographical units to present the inherent spatial difference and tendency. The spatial autocovariate variable has the same unit of the proportion of SBI. As shown in Figure 3, the spatial correlation of SBI has a strong spatial tendency and heterogeneity, which presents a transitional and gradual change throughout the years in Ethiopia. The red colors in Figure 4 showed that the spatial correlation is very high consistently in Liben and siti in Somali region, east and west harerge in Harari region and awi zone in Amhara region over the EDHS years of 2000, 2005, and 2011. And the spatial correlation of SBI is high in doolo and Korahe zones of Somali region in 2016 (Figure 4).

The final autologistics regression model reported a significant positive association between women’s Residence with SBI of women across all years (2000–2016). After controlling for other variables in the model, women who resided in rural areas were 38.4% [OR = 1.384, CI (1.16–1.651)], 42.3% [OR = 1.423, CI (1.18–1.717)], 48.3% [OR = 1.483, CI (1.26–1.744)], and 55.4% [OR = 1.554, CI (1.317–1.835)] more likely to have short birth intervals as compared to those from the urban area in 2000, 2005, 2011, and 2016, respectively. Women who breastfed their child for more than 24 months were 24% [OR = 0.859, CI (0.765–0.964)], 32.6% [OR = 0.674, CI (0.613–0.752)], 20.6% [OR = 0.794, CI (0.721–0.874)], and 31.8% [OR = 0.682, CI (0.618–0.752)] less likely to have short birth intervals than those who breastfed for less than 6 months in 2000. 2005, 2011, and 2016 EDHS years, respectively, while controlling for other variables in the model (Table 3).

**Table 3:** Parameter estimates for autologistic regression model in 2000–2016.

Year		2000			2005			2011			2016		
Parameter	Categories	AOR	95% CI		AOR	95% CI		AOR	95% CI		AOR	95% CI	
Intercept		0.863			1.567			1.172			1.015		
Region	Addis	1.303	0.989	1.717	0.910	0.687	1.206	0.965	0.727	1.281	1.251	0.947	1.654
	Afar	^**^1.406	1.093	1.810	^*^1.321	1.006	1.736	1.111	0.861	1.432	^**^1.825	1.416	2.352
	Amhara	0.937	0.775	1.134	^**^0.702	0.579	0.852	^*^0.803	0.667	0.966	0.858	0.700	1.052
	Ben-Gumz	1.104	0.881	1.384	0.846	0.661	1.082	1.085	0.873	1.348	^**^1.422	1.134	1.783
	Dire Dawa	^**^1.585	1.173	2.140	0.895	0.644	1.243	^*^1.342	1.010	1.784	^**^1.708	1.304	2.239
	Gambella	^**^0.630	0.484	0.821	^**^0.590	0.450	0.774	^*^0.732	0.562	0.953	0.881	0.676	1.146
	Harari	^**^1.705	1.269	2.292	1.067	0.802	1.420	^**^1.626	1.255	2.107	^**^1.592	1.219	2.078
	Oromiya	^**^1.369	1.122	1.670	0.985	0.804	1.206	^**^1.346	1.103	1.643	^**^1.500	1.216	1.852
	SNNP	1.090	0.885	1.342	^*^0.749	0.599	0.936	1.049	0.841	1.310	^*^1.256	1.000	1.577
	Somali	^**^2.135	1.615	2.823	1.189	0.901	1.569	^**^1.736	1.339	2.250	^**^2.783	2.166	3.575
	Tigray(Ref)												
Residence	Rural	^**^1.384	1.160	1.651	^**^1.423	1.180	1.717	^**^1.483	1.261	1.744	^**^1.554	1.317	1.835
	Urban(Ref)												
Mother education	Higher	0.660	0.352	1.238	0.944	0.565	1.577	0.811	0.539	1.221	1.316	0.952	1.818
	Primary	0.981	0.837	1.151	1.100	0.946	1.280	^**^0.831	0.737	0.937	0.989	0.875	1.118
	Secondary	^**^0.727	0.570	0.928	0.915	0.714	1.172	0.752	0.556	1.018	1.115	0.884	1.407
	No education(Ref)												
Religion	Moslem	0.984	0.777	1.247	0.935	0.708	1.234	0.977	0.699	1.367	0.949	0.678	1.327
	Orthodox	^*^0.776	0.614	0.980	^**^0.662	0.501	0.875	^**^0.767	0.648	0.908	^**^0.632	0.449	0.888
	Protestant	0.818	0.641	1.044	0.967	0.733	1.276	1.072	0.908	1.264	0.812	0.581	1.135
	Others(Ref)												
Type of the index child	Multiple	1.022	0.707	1.476	0.818	0.537	1.245	0.810	0.573	1.146	^*^0.640	0.443	0.925
	Single(Ref)												
Sex of the index child	Female	0.961	0.880	1.049	1.010	0.920	1.108	0.951	0.870	1.039	1.013	0.925	1.109
	Male(Ref)												
Survival status of the index child	Alive	^**^0.608	0.525	0.704	^**^0.634	0.526	0.763	^**^0.615	0.509	0.743	^**^0.667	0.540	0.823
	Dead(Ref)												
Husband education	Higher	0.992	0.690	1.427	0.779	0.540	1.125	0.889	0.659	1.199	0.909	0.717	1.151
	Primary	^**^1.317	1.163	1.490	1.050	0.924	1.194	1.067	0.959	1.188	1.030	0.913	1.161
	Secondary	^**^1.291	1.078	1.545	0.840	0.699	1.009	0.946	0.765	1.170	0.951	0.782	1.156
	No education(Ref)												
Access of mass media	No	^*^1.133	1.008	1.274	0.943	0.840	1.059	1.099	0.993	1.215	0.901	0.802	1.013
	Yes(Ref)												
Breastfeeding practice	Yes	^*^0.859	0.765	0.964	^*^0.679	0.613	0.752	^**^0.794	0.721	0.874	^**^0.682	0.618	0.752
	No(Ref)												
Mothers occupation	has work	0.955	0.866	1.054	0.930	0.839	1.031	0.941	0.854	1.036	0.910	0.823	1.005
	No work(Ref)												
Contraceptive use	Use	1.058	0.896	1.250	0.959	0.831	1.108	1.035	0.922	1.162	^**^0.878	0.785	0.982
	Not use(Ref)												
husband occupation	Has work	1.127	0.750	1.693	1.035	0.707	1.513	^**^0.631	0.475	0.839	0.982	0.841	1.146
	No work(Ref)												
Marital status	Married	1.119	0.983	1.274	1.077	0.931	1.247	^*^1.201	1.043	1.384	^*^1.257	1.018	1.552
	Single	0.569	0.203	1.590	0.434	0.145	1.300	0.708	0.275	1.820	0.959	0.441	2.085
	Others(Ref)												
Wealth index	Middle	1.116	0.976	1.277	0.979	0.850	1.127	0.932	0.818	1.062	^**^0.817	0.709	0.943
	Rich	0.979	0.882	1.087	1.112	0.972	1.273	1.010	0.891	1.146	^**^0.770	0.672	0.882
	Poor(Ref)												
Spatial effect		^*^0.551	0.332	0.916	^**^0.431	0.273	0.682	^*^1.377	1.006	1.885	^*^0.660	0.442	0.985

AOR, Adjusted Odds ratio; *Significant at value of *p* = 5%; **Significant at value of *p* = 1%.

The survival status of the index child has a significant association with SBI in all EDHS years (2000–2016). The estimated odds of SBI were 0.608 [OR = 0.608, CI (0.525–0.704)], 0.634 [OR = 0.634, CI (0.526–0.763)], 0.615 [OR = 0.615, CI (0.509–0.743)], 0.667 [OR = 0.667, CI (0.540–0.823)] times lower for women whose second most recent child was alive compared to women whose second most recent child was dead in 2000, 2005, 2011 and 2016, respectively, while controlling for other variables in the model (Table 3).

The autologistic model confirms the spatial correlation of SBI between zones. The spatial autocorrelation variable with SBI was a negative value, −0.5952, −0.8417, and −0.4161 which indicates that zones with a low proportion of SBI were usually surrounded by zones with a high proportion of SBI in 2000, 2005, and 2016 EDHS years, respectively. Whereas the spatial autocorrelation variable with SBI was a positive value, 0.3197 in the 2011 EDHS year, which indicates zones with a lower proportion of SBI were usually surrounded by zones with a lower proportion of SBI and that zones with a higher proportion of SBI were usually surrounded by zones with a higher proportion of SBI. assuming that the other variables are held constant (Table 3).

By introducing the spatial autocovariate variable, the contribution of the constant is reduced significantly in the autologistic model in all EDHS years (2000–2016). It changes from OR = 1.050 (for logistic regression) to OR = 0.863 (for autologistic regression), from OR = 2.225 (for logistic regression) to OR = 1.567 (for autologistic regression), from OR = 1.409 (for logistic regression) to OR = 1.172 (for autologistic regression), from OR = 1.211 (for logistic regression) to OR = 1.015 (for autologistic regression) in 2000, 2005, 2011, and 2016 EDHS, respectively. As the result, the spatial autocovariate variable can be comprehended as the spatial inherent residual to reflect spatial effect in space data, which can reduce bias in health risk assessment. The binary logistic regression model’s inherent residual errors were reduced due to the spatial autocovariate variable (Table 3).

## 4. Discussion

Using EDHS data from 2000 to 2016, this study aims to evaluate the distribution and related variables of SBI in the Ethiopian administrative zone using the autologistics spatial analysis approach. The spatial analysis has consistently shown hot and cold spots areas of SBI among women in Ethiopian administrative zones. The findings of this study show that residence, survival status of index child, religion and breastfeeding practice are consistent determinants of SBI of women in all EDHS years (2000–2016). The findings also indicated the spatial effect through the autocovariate variable(si) was a determinant of SBI across all EDHS years (2000–2016). Moreover, the mother’s education, husband’s education level, and access to mass media were determinants of SBI of women in 2000 EDHS year. Mothers’ education, husband occupation, and marital status were determinants of SBI of women in 2011 EDHS year and types of index child, marital status, and contraceptive use were determinants of SBI of women in 2016 EDHS year.

The decrease in the mean short birth interval observed during the 2005–2016 Ethiopian Demographic and Health Survey (EDHS) could be due to a combination of factors, including changes in health policies and socio-economic determinants. One possible explanation could be the impact of family planning programs and policies implemented during this period. The Ethiopian government has implemented various programs and policies aimed at increasing access to family planning services and promoting the use of modern contraceptive methods. These efforts may have contributed to a decrease in short birth intervals by reducing unintended and closely spaced pregnancies.

In addition to changes in health policies, socio-economic factors such as increased educational attainment, improved access to healthcare services, and changes in social norms and attitudes toward family size and childbearing may have also contributed to the decrease in short birth intervals. For instance, women who have access to education and healthcare services may be more likely to delay childbirth and plan their pregnancies, leading to longer birth intervals. Overall, the decrease in the mean short birth interval observed during the 2005–2016 EDHS could be attributed to a combination of health policies and socio-economic determinants. Further analysis and research would be necessary to identify the specific factors that have contributed to this trend.

The spatial analysis indicates that the distribution of short birth intervals was clustered across the Ethiopian administrative zones across all EDHS years (2000, 2005, 2011, and 2016) which implies the clustering of similar values (high values are found closer together, and low values are found closer together) of the SBI among neighboring observations. The Fafan zone in the Somali Region and the Harari and East Harerge zones in the Harari Region were consistently hotspots of SBI in all the survey years. Moreover, West Harerge, Bale, Guji, Borona, and Gedio were hotspot zones in 2000. The Sitti zone in Somalia region and Zone 3 in the Afar Region were hotspot zones in 2005. West Harerge, Zone 3, Arsi, Borona and Segen are high-risk zones in 2011 EDHS year. Siti, Jarar, Korahe, Shebelle and Afder of Somali region and East and West Harerge in the Oromia Region are hotspot zones in 2016. This finding is consistent with two studies conducted in Ethiopia (16, 17).

In this study, women who had never used contraceptive methods preceding the conception of the last child were more likely to be at risk of having an SBI compared to women who had used contraceptive methods in 2016. This finding is consistent with the findings of studies by Gebrehiwot et al. (7) and Ejigu et al. (9) that state that participants who had never used the contraceptive method before having their last child were more likely to be at risk for having an SBI than the participants who used contraceptive methods. This is clear that the purpose of the contraceptive method is either to limit or space births.

Our study indicated that place of residence has a significant association with an SBI in all the EDHS years (2000–2016), i.e., women who resided in rural areas were more likely to have short birth intervals as compared to those from urban areas. This finding is supported by Yohannes et al. (18) and Exavery et al. (19), who found that rural women were more likely to have SBIs than urban women. Similarly to the study by Exavery et al. (19), who found that formerly married women (divorced or widowed) were less likely to space their births poorly compared to married women, our study found that the prevalence of SBIs was higher for married women compared to divorced and widowed women. This suggests that women who are currently not married may have less opportunity for childbearing and are consequently likely to have longer inter-birth intervals than married women.

The prevalence of SBI among mothers who lost their index child was higher compared to women whose index child was alive for all EDHS years (2000–2016). This finding was consistent with evidence from studies conducted in Bangladesh (20, 21) that found that the experience of a child dying at birth in a woman’s second recent pregnancy was a significant predictor of SBI. The mother’s education level significantly influenced the SBI of women. This implies that women who had more education were less likely to have an SBI compared to women who had no education in 2000 and 2011. Similarly to this study, studies conducted in Ethiopia by Khan et al. (22) and Kozuki et al. (23) showed that the odds of having a short inter-birth interval were higher among mothers who had no formal education as compared to their women counterparts who attended formal education.

Studies conducted in Ethiopia by Ejigu et al. (9) and Kemi and Joseph (20) found that mothers who breastfed the preceding child for less than 24 months were more likely to have short interbirth intervals than mothers who breastfed for 6 months. Similarly, the current study showed that women who breastfed their child for more than 24 months were 31.8% less likely to have an SBI than those who breastfed for less than 6 months in all the EDHS years (2000–2016).

Mothers with a richer wealth index were 23% less likely short birth intervals than women with a poorer wealth index in 2016. This finding is consistent with evidence from a study done in Ethiopia (19) which stated that the odds of having a short interbirth interval were higher for mothers who belonged to the poorest wealth index than the richest group of mothers.

In this study, husbands’ education and access to mass media were significantly associated with an SBI in 2005. Women whose husbands had a primary education level and secondary education level were 31.7% and 29.1% more likely short birth intervals, respectively, as compared to women whose husbands have no education. This finding is consistent with evidence from a study done by Chernet et al. (24).

The findings of the study indicated religion was significantly associated with an SBI across all the EDHS years (2000–2016). Women who identified as part of the Orthodox religion were less likely to have an SBI compared to women who identified as Catholic, Traditional, or with other religions. This finding is consistent with evidence from a study done by Gebrehiwot et al. (7). In line with a study done by Exavery et al. (19), our study showed that mothers with multiple-index children were 36% less likely to have an SBI as compared to mothers with single-index children.

According to the EDHS in 2000, 2005, and 2016, the spatial variable had a negative significant effect, while in 2011 the spatial variable has a positive significant effect. This research shows that zone with a low proportion of SBI was usually surrounded by zones with a high proportion of SBI in 2000, 2005, and 2016, while zones with a lower proportion of SBI were usually surrounded by zones with a lower proportion of SBI in 2011 EDHS. In this study, the standard errors of the estimated coefficients were underestimated in the model that ignored spatial effects compared with the model that adjusted for spatial effects.

## 5. Conclusion

This study found that the spatial distribution of the proportion of SBI in Ethiopia depends on characteristics related to the demographic, socioeconomic, reproductive, behavioral, and child status variables. The prevalence of SBI in Ethiopia has slightly decreased from 47.5% to 44%. However, the reduction in SBI was not homogeneous in the zones over time. In the 74 zones of Ethiopia, we found variations over the 2000–2016 survey years in the distribution of SBI among women. In this study, we concluded that the spatial distribution of SBI was found significantly clustered in particular Ethiopian administrative zones. This study showed that high-risk areas of SBI were found consistently in the Fafan zone of the Somali Region and the Harari and East Harerge zones of the Harari Region in all the survey years. The autologistic model that adjusted for spatial effects performed better and showed that there was a significant spatial correlation of SBI between zones with a low proportion of SBI that were usually surrounded by zones with a high proportion of SBI and vice versa for the years 2000, 2005, and 2016. Moreover, zones with a lower proportion of SBI were usually surrounded by zones with a lower proportion of SBI, and zones with a higher proportion of SBI were usually surrounded by zones with a higher proportion of SBI in 2011. This study identified the death of the index child, residing in rural areas, and having no breastfeeding practice as the risk factors that increase the risk of an SBI. In contrast, having a breastfeeding practice, residing in urban areas, and the survival of the index child were consistent risk factors that decrease the SBI in all the EDHS years (2000–2016). Thus, we recommend that interventions should give special attention to focusing on mothers whose index child has died, who reside in rural areas, and who do not breastfeed their children.

## Data availability statement

The raw data supporting the conclusions of this article will be made available by the authors, without undue reservation.

## Ethics statement

The studies involving human participants were reviewed and approved by Bahir Dar University College of Science Ethics Committee. The patients/participants provided their written informed consent to participate in this study.

## Author contributions

GA and AM designed and drafted the manuscript and analyzed and interpreted the results. HF and AM participated in the design of the methodology, data analysis and critically read the manuscript, and gave constructive comments for the development of the manuscript. All authors contributed to the article and approved the submitted version.

## Conflict of interest

The authors declare that the research was conducted in the absence of any commercial or financial relationships that could be construed as a potential conflict of interest.

## Publisher’s note

All claims expressed in this article are solely those of the authors and do not necessarily represent those of their affiliated organizations, or those of the publisher, the editors and the reviewers. Any product that may be evaluated in this article, or claim that may be made by its manufacturer, is not guaranteed or endorsed by the publisher.

## Footnotes

^1^ https://dhsprogram.com/
